# Selecting treatment method for ovarian masses in children – 24 years of experience

**DOI:** 10.1186/s13048-017-0353-0

**Published:** 2017-09-11

**Authors:** Justyna Łuczak, Maciej Bagłaj

**Affiliations:** Pediatric Surgery and Urology Department, Wroclaw Medical University, 52 M. Sklodowskiej Curie ST, 50-369 Wroclaw, Poland

**Keywords:** Ovarian neoplasms, Ovarian masses, Child, Management algorithm

## Abstract

**Background:**

Epidemiology and pathology of ovarian tumors in the pediatric population are very different of these encountered in women. Few attempts have been made to analyze the whole spectrum of ovarian pathology in children, and only some of them included series of more than 200 cases. We performed a retrospective analysis of clinical and diagnostic aspects of ovarian tumors and tumor-like lesions in girls in order to identify the characteristics associated with malignancy with an attempt to elaborate a clinical management algorithm.

**Results:**

The study group comprised 214 patients operated on for ovarian tumor in years 1991-2014 at the pediatric surgical center. Non-neoplastic ovarian lesion was diagnosed in 127 females. Sixty-five patients had a benign tumor and 22 had a malignant lesion. Abdominal pain was the most common symptom in the non-malignant lesion group. Patients with ovarian malignancy presented predominantly with abdominal distension and palpable mass. In the non-malignant group imaging studies revealed cystic lesion in 124 patients (68.89%) and solid mass in 10 (5.55%). Malignant lesion showed a solid or mixed structure in all cases. Positive tumor markers were noted in 14 (13.7%) patients with a benign lesion and in 14 (70%) with ovarian malignancy. Large lesions were found in 77.3% of girls with a malignant mass, while only in 32.8% of patients with a benign lesion (*p* < 0.001). In the group of solid tumors positive tumor marker results occurred more frequently in patients with diagnosed malignant tumors (*p* < 0.05). Positive tumor markers, large size of the lesion and age below 14 years were independent variables differentiating malignant tumors from non-malignant lesions (*p* = 0.00000).

**Conclusions:**

Predominantly solid structures noted on imaging studies, large dimension and positive tumor markers are clinical predictors of malignancy. A diagnosis of purely cystic lesions with negative markers or of a small size should be an indication for a gonad-sparing procedure. Treatment guidelines for ovarian lesions in children should be established on the basis of multicenter prospective studies and introduced as soon as possible in order to improve and unify the ovarian preservation rates across the pediatric surgical centers.

## Background

Ovarian masses in girls represent a wide pathological spectrum ranging from tumor-like conditions to highly aggressive malignant tumors. Depending on clinical presentation, young patients with ovarian lesions are admitted initially for evaluation to pediatric or pediatric surgical centers. Most lesions require eventually an operative management [1–5]. Older girls may be referred to the adult gynecological departments thus escaping the pediatric surgical database. It renders objective epidemiological studies in pediatric population very difficult. Few attempts have been made to analyze the whole spectrum of ovarian pathology in children, and only some of them included series of more than 200 cases [2, 3, 6–9]. Management of ovarian lesions varies with demographic, hospital, and physician factors. International treatment guidelines dedicated to children are still not established, causing a great difficulty in making appropriate therapeutic decisions (a search of PubMed: English language; 1966–2017; search terms: “ovarian neoplasms” and “child”/ “ovarian masses” and “child”). This in turn poses a threat to the patient’s life and fertility in the future. It is essential to understand true clinical nature of ovarian lesions in pediatric population and to elaborate a clinical management plan suited for young patients.

Therefore, we performed a detailed analysis of clinical aspects of ovarian masses in girls operated on at our institution. Based on the 24-year experience we aimed to determine the features that are associated with gonadal malignancy and to develop management algorithm useful in clinical practice.

## Methods

We analyzed retrospectively medical files of all consecutive patients aged 0-18 who underwent surgical procedures for ovarian lesions between 1991 and 2014 at the University Department of Pediatric Surgery and Urology in Wroclaw, Poland. Patients with a paraovarian cyst and those with ovarian torsion without an accompanying lesion were excluded. The demographic data, presenting symptoms and signs, results of laboratory and diagnostic studies (including ultrasound examination, additional imaging studies and tumor markers), details of surgical procedures, and clinical outcomes (including preservation rate), were extracted in each case. Ovarian mass characteristics were evaluated by preoperative imaging (structure and size) or by description of the procedure (size). An ovarian lesion was described arbitrarily as large when its diameter was 10 cm or more in girls aged between 1 and 18 years and 5 cm or more in newborns and infants. Such classification was based on the previous experience of other authors, in order to obtain comparable results [3, 6, 10, 11]. A choice of an operative technique (either laparoscopic or open) depended solely on a surgeon’s preference. The extent of gonadal resection was based on intraoperative findings and it ranged from total, when the whole gonad affected by lesion was removed, to partial resection, when at least the remnant of ovarian tissue was preserved. Preservation rates were compared taking into consideration the operative method, the histological type, the size of the mass, the presence of ovarian torsion and the study period (1991-2002 vs 2003-2014). A final diagnosis was made on the basis of a pathology report. All clinical characteristics were reviewed to test their association with malignancy. Therefore, the study group was divided into two subgroups of patients; girls with tumor-like lesions combined with benign tumors (non-malignant group) and malignant tumors.

Parameters in groups were expressed as median and quartiles or as mean and standard deviation. The statistical significance between means for different groups was calculated with the non-parametrical U Mann-Whitney test. The statistical significance between frequencies was calculated by the chi-square test χ [2]_df_ with Yates correction with corresponding degree of freedom df (df = (m-1)*(n-1), where m – number of rows, n – number of columns). For significant frequencies Cramer’s contingency coefficient was calculated. Multivariate logistic regression analysis was performed to estimate the model discriminating patients with malignancy from patients with non-malignant lesions. A *p* value of less than 0.05 was required to reject the null hypothesis. Statistical analysis was performed using the EPIINFO Ver. 3.5.2 (17-12-2010) and SPSS Statistics 20 (IBM) software packages.

The study was approved by the Ethical Committee of the Medical University of Wroclaw.

## Results

### Epidemiology and presentation

The study group comprised 214 patients. The mean age was 11.1 ± 5.7 years. Non-malignant mass was noted in 192 girls, including 127 girls (59.35%) with non-neoplastic lesions and 65 girls (30.37%) with benign tumors. Twenty-two patients (10.28%) presented with malignant tumors. There was no significant statistical difference between the non-malignant and malignant group when age at presentation was factored into the analysis [median age in non-malignant group was 14.0 (8.0÷16.0) years versus 10.0 (7.0÷14.0) years in malignant group, *p* = 0.134]. However, logistic regression analysis revealed the age below 14 years to be one of the independent variables differentiating malignant tumors from non-malignant lesions (*p* = 0.0270). Mature teratoma was the most frequent lesion among benign neoplasms. The most common malignant lesion noted in our study was dysgerminoma. The histological distribution of all ovarian lesions is presented in the Table 1.

**Table 1:** The histological distribution of ovarian lesions in the study group

Type Of The Lesion			Number Of Patients	Median Age (Lower÷Upper Quartile)
Non-Malignant Lesions	Tumor-Like Lesions	Simple cyst	69	14.0 (8.0÷16.0) years
		Hemorrhagic cyst	55	
		Endometriosis	3	
	Benign Tumors	Mature teratoma	49	
		Cystadenofibroma	7	
		Serous cystadenoma	5	
		Mucinous cystadenoma	4	
Malignant Lesions		Dysgerminoma	7	10.0 (7.0÷14.0) years
		Juvenile granulosa cell tumor	4	
		Immature teratoma	4	
		Burkitt’s lymphoma	2	
		Malignant mesenchymoma	1	
		Embryonal carcinoma	1	
		Serous papillary carcinoma	1	
		Androblastoma	1	
		Sertoli cell tumor	1	

**Fig. 1:**
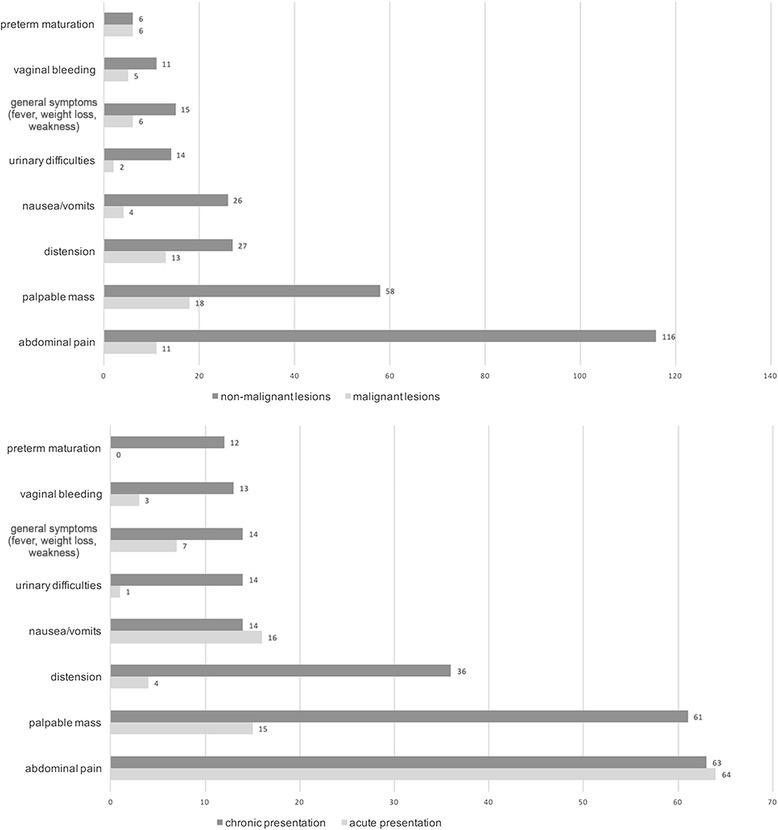
The most common presented symptoms

There were 147 patients (68.69%) with chronic presentation (no symptoms requiring immediate surgical intervention). Sixty-seven girls (31.31%) presented with acute symptoms (admitted to the hospital as emergency due to relevant pain or/and vomiting, weakness, fever). Abdominal pain, palpable mass, distension, nausea and vomiting were the most frequent clinical features noted in the whole study group. Abdominal pain predominated in the non-malignant group and was noted in 116 of them (60.42%), while palpable mass was the most frequent symptom in girls with malignant tumors (81.82%). Palpable mass was detected only in 15 girls (22.39%) with acute presentation. Chronic presentation was associated with pain in 63 girls (42.86%) and with palpable mass in 61 (41.50%); Fig. 1. There were 2 malignant lesions in the group of patients with acute presentation.

### Diagnostic studies

Abdominal ultrasound scan (US) showed a cystic structure in 124 girls with non-malignant lesion (68.89%). A heterogeneous ovarian lesion was noted in 46 (25.55%) and a solid mass in further 10 (5.29%) girls from this subgroup. In the malignant group, 10 girls presented with a mixed solid-cystic tumor (52.63%). In 4 of them ovarian lesions were predominantly cystic with peripheral solid portion or septation. In further 9 girls (47.39%) a solid structure of the mass was found.

In the whole study group, 52 girls (24.29%) had computed tomography (CT) or magnetic resonance imaging (MRI) studies performed preoperatively. Twenty-six girls had a large tumor (with a diameter of more than 10 cm). In 13 patients this additional examination led to the evaluation of tumor origin or its real structure changing the initial radiological description of the lesion (based on US scan). Ten of the 13 lesions were large. Statistical analysis revealed a relationship between the size of the lesion and the occurrence of a change of the radiological description (*χ* ^2^_2_ = 5.09; *p* < 0.05). That might indicate usefulness of diagnostic imaging extension in these cases.

The results of tumor markers evaluation (AFP – alpha-fetoprotein, β-hCG - beta subunit of human chorionic gonadotropin, CA125 - cancer antigen 125, LDH - lactate dehydrogenase) were available for 122 girls (57.01%). They were elevated in 14 girls (13.72%) with the non-malignant mass. In all of them only one marker was positive, being CA-125 in 11 of them, LDH in 2 and AFP in one patient. In 2 girls with ovarian malignancy data regarding markers were missing, while in six of them markers were negative. Fourteen patients (70.00%) had positive markers. Two markers were elevated in 6 patients and one marker was positive in 8 patients. AFP and CA-125 were elevated in 5 patients each. Four girls had β -hCG positive and in further 6 LDH was found elevated (Table 2).

The Chi square test revealed that there is a significant association between the type of structure of the lesion seen on US, tumor markers and its final pathology. In the group of solid tumors, analysis revealed that positive tumor marker results occur more frequently in patients with diagnosed malignant tumors. The opposite relation was found in the group of non-malignant lesions (*χ* ^2^_2_ = 6.74; *p* < 0.05). The relationship between these variables was strong, with the measure of Cramer’s coefficient of 0.60.

### Large lesions

In 80 cases (37.38%) the tumor was defined as large (based on the results of preoperative imaging studies and intraoperative findings). Large lesions were more frequent in the group of malignant tumors and appeared in 17 of them (77.27%) (*χ* ^2^_2_ = 16.67, *p* < 0.001). In the benign and non-neoplastic lesion group they were noted in 32.81% of cases. Among 28 girls with positive markers, 19 (67.86%) had a large ovarian mass (Table 2).

**Table 2:** The results of tumor markers evaluation

	*Histological type*				
		Tumor-like lesion	Benign tumor	Malignant tumor	Summary
*marker*	AFP	0	1	5	6
	β-hCG	0	0	4	4
	CA125	7	4	5	16
	LDH	1	1	6	8
	Large Lesion and Positive Markers*	1/57 (1.75%)	6/45 (13.33%)	12/20 (60.00%)	15.57%
	* (among patients with markers tested)				
	Solid Lesion and Positive Markers	1/56 (1.78%)	0/45 (0%)	5/18 (27.78%)	5.04%
	*(among patients with markers tested and US performed)				

### Treatment

One–hundred and seven girls (50%) had laparotomy and 107 (50%) had laparoscopy performed as an initial operative approach. Conversion to open procedure was noted in 18 girls (8.41%). In the second part of our study, since 2003, the number of patients operated on with laparoscopy increased to 55.00%. All girls, but one, with malignant tumor were subjected to formal laparotomy. They all underwent at least complete resection of the affected gonad and therefore were excluded from the analysis concerning ovarian tissue preservation.

Ovarian tissue sparing technique (preservation of the ovarian tissue of the affected gonad) was applied in 55.66% patients operated on with laparoscopic technique and in 17.44% of girls in whom open procedure was performed. Regarding the final histology, gonad-sparing procedure was recorded in 48.03% of patients with non-neoplastic lesions and only in 18.46% of those with benign tumors (Table 3). Formal open approach was chosen in 48 patients (76.19%) with large ovarian masses. The percentage of large lesions was higher in the group of benign tumors. The preservation rate of ovarian tissue in large lesions was 17.46% compared to 48.84% in the remaining group.

**Table 3:** Ovarian tissue preservation rates in selected group of patients

	Overall	Operation Method		Histological type of The lesion		Large Lesion	Torsion	
		Laparoscopy	Laparotomy	Tumor-like	Benign		˗	+
*Preservation rate*	38.54% (36.03% 2003-2014 vs 28.30% 1991-2002)	55.66% (14.02% of large lesions)	17.44% (46.15% of large lesions)	48.03% (22.05% of large lesions)	18.46% (53.85% of large lesions)	17.46%	40.82%	22.22%

The relationship between the extent of gonadal resection and operative method was less relevant in infants. In this subset of patients, gonadal tissue preservation was noted in 8.33% of cases subjected to laparoscopy and in 22.22% of those operated on with open technique. Moreover, tissue sparing was possible in 10.53% of those with large lesions and 9.09% of girls with lesions not classified as large.

The analysis revealed that the preservation rate increased in the second half of our study (2003-2014) when compared to the first study period between 1991 and 2002 (36.03% vs 28.30%).

### Factors excluding malignancy

Factors like pure cystic structure on ultrasound scan and negative tumor markers were found in 54 patients and none of them had a malignant mass diagnosed (in this group all factors were obtained in each girl). Statistical analysis (logistic regression) revealed that positive tumor markers, large size of the lesion and age below 14 years are interdependent variables differentiating malignant tumors from non-malignant lesions (*χ* ^2^_3_ = 49.8, *p* = 0.00000; positive markers – *p* = 0.00000, large size – *p* = 0.0108, age < 14 years – *p* = 0.027). There was no case of a malignant mass in 32 patients with clinical features mentioned above (all factors obtained in each girl). Summary of clinical data in the two groups of patients with non-malignant and malignant lesion is presented in the Table 4.

**Table 4:** Comparison of the clinical data in the non-malignant and malignant lesion group

	Malignant	Non-malignant	*p* value
*Number of patients*	22	192	
*Age(years)*	10.0 (7.0÷14.0)	14.0 (8.0÷16.0)	0.134
0-1	0	30/ 15.63%	
2-4	3/ 13.64%	5/ 2.60%	
5-8	7/ 31.82%	19/ 9.90%	
9-14	6/ 27.27%	63/ 32.81%	
15-18	6/ 27.27%	75/ 39.06%	
<14 years	16	94	0.027
>14 years	6	98	
*Symptoms*			
abdominal pain	11/ 50%	116/ 60.42%	
palpable mass	18/ 81.82%	58/ 30.21%	
distension	13/ 59.9%	27/ 14.06%	
*Us Result*	19/ 86.36%	180/ 93.75%	
solid	9/ 47.37%	10/ 5.55%	<0.05 (in association with positive tumor markers)
complex	10/ 52.63%	46/ 25.55%	
cystic	0/ 0.00%	124/ 68.89%	
*Size Of The Lesion*			<0.001
large lesion	17/ 77.27%	63/ 32.81%	
lesion that was not described as large	5/ 22.73%	129/ 67.19%	
*Bilateral Lesion*	3/ 13.64%	11/ 5.73%	
*Tumor Markers*			<0.00000
positive	14/20 (70.00%)	14/102(13.72%)	
negative	6/20 (30%)	88/102(86.28%)	

## Discussion

Ovarian tumors in children and adolescents are predominantly a subject of clinical reports from pediatric surgical centers as most patients from this age group require eventually surgical management [2, 3, 6–9]. For many years the therapeutic principles elaborated for adult patients were applied by pediatric surgeons too. However, epidemiology of ovarian tumors in pediatric population differs to a large extent from that of women so such direct transfer of management approach seems nowadays unwarranted [2, 5, 12–14]. Only better understanding of prepubertal ovarian biology and natural history of its pathology may help to elaborate effective and safe diagnostic and therapeutic strategies for girls. Unfortunately, paucity of clinical reports dedicated exclusively to children, including all types of ovarian lesions and comprising a large cohort of patients makes an objective insight into these aspects difficult.

Our 24-year retrospective study included all girls with ovarian lesion referred to the university pediatric surgical department. It may therefore reflect the true epidemiology of these tumors as in the Lower Silesia (a region in Poland) the majority of girls aged up to 18 years were admitted to the pediatric centers. The retrospective design poses limitations. It forces the reliance on documentation of patient symptoms by the charting physician. In addition, the particular intraoperative decisions of the surgeons were not clear. Nevertheless, the study represents a relatively large group of patients.

It is of no surprise that in our surgical series of patients, those with tumor-like pathology predominate. It confirms the observation of other authors reporting their clinical experience. An incidence of malignancy among pediatric patients is low but this is the most relevant aspect of ovarian pathology [13, 15, 16]. However, we noted some contradictory results regarding age distribution among girls with malignant tumors in the literature [13, 15, 17]. Brookfield. et al., in a large group of 1037 patients noted that most girls with malignant lesions were between 15 and 19 years of age, while Oltman et al. in a group of 424 patients found the highest incidence of malignancy between 1 and 8 years of age [6, 16]. In our study we noted the mean age of 10.25 years in malignant lesion group and the highest incidence was in the group aged 5-9 years. Logistic regression analysis revealed age below 14 years to be one of the interdependent variables differentiating malignant tumors from non-malignant lesions. A small number of malignant lesions in our series and noted by other authors dictate the need for multicenter collaborative study aimed to have an objective insight into epidemiology of ovarian tumors in children.

As regards clinical presentation of ovarian pathology in children we noted another discrepancy between various clinical series in the literature. Similar to former studies abdominal pain was the major complaint but there is a difference in frequency of other symptoms, including those which should be easily observed by the parents like visible abdominal mass or distension [3, 7]. In our series of patients with malignancy, more than 80% presented with palpable mass. At the time of initial examination, it was diagnosed as large in 77% of them. Such clinical presentation may indicate significant delay in seeking of medical consultation by the patients and their parents. Surprisingly in a series of girls reported by Papic et al. only 28% girls with malignant ovarian lesions presented with palpable mass although 89% of them had lesion of size >10 cm^15^. There is no strict correlation between the size of ovarian mass and its histology but analyzing the data from the literature it appears that most malignant lesions are larger than 8-10 cm. Only a few reviews provide precise data for malignant and non-malignant lesions separately but there is an evident difference between both groups of patients in terms of size of an ovarian mass [3, 6, 10, 11, 18, 19]. A large ovarian mass must therefore always raise a concern and should be treated as a risk factor for malignancy [6, 8, 11].

Imaging studies play an important role in the evaluation of girls with ovarian pathology. US scan is the study of choice during initial assessment. It provides relevant data although in case of large or complex tumors it may not be specific [20–24]. The need for further imaging beyond ultrasound in other benign ovarian diseases is not clear. However, additional imaging techniques are valuable if, clinical evaluation and ultrasound examination suggest possible ovarian malignancy. In some patients it is difficult to localize the origin and the extent of the lesion and the diagnostic work-up of such indeterminate masses should include magnetic resonance imaging or CT (the latter primarily reserved for tumor staging). However, the MR imaging examination is longer and may require sedation in a younger child. Availability of pediatric sedation and the radiologist’s confidence in interpreting the imaging examination might influence the decision to use CT or MRI [3, 10, 18, 20, 21, 23–25]. Time is a critical factor in case of ovarian torsion, and early surgical intervention may be able to save a viable gonad. As CT and MRI have not proven useful in the detection of torsion, ultrasound is the imaging modality of choice. Ovarian torsion is infrequently associated with a diagnosis of malignant neoplasm [21, 26]. Therefore, unless there is any suspicion of malignancy, in the setting of acute clinical symptoms and the suspicion of ovarian torsion, the surgical treatment should not be delayed.

Apart from the size, the structure of the tumor and its characteristics are very important in preoperative evaluation. Reviewing the data of other authors and our own series it may be concluded that a solid lesion must always be viewed as potentially malignant. In our series none of the malignant lesions was purely cystic but such cases were reported by other authors [4].

Evaluation of tumor markers is an important step in a preoperative assessment of a girl with a pelvic mass. Although many studies have confirmed association of their elevated levels with malignancy, there are some that highlight their limited diagnostic accuracy [6, 27, 28]. Our results revealed their positive role in predicting histology of ovarian lesions, especially when coupled with their features on imaging studies. Specifically, AFP and β-hCG were elevated in all but one patients with malignant lesion only. The same observation was made by Papic et al. Oltman et al. found only one patient with a benign mass and positive AFP [6, 15]. Our experience shows clearly that LDH and CA-125 are not very specific as these were the positive markers in non-malignant lesions too. But when positive markers are correlated with the size and structure of the affected ovary, they provide very important prognostic value.

The results obtained by us clearly indicate that a purely cystic lesion of the ovary smaller than 10 cm and not associated with positive tumor markers should be regarded as a benign lesion and therefore should be an indication for gonad-sparing minimally invasive surgery. Conversely, a large ovarian lesion with solid components and with positive markers should be viewed as potentially malignant and treated respectively.

**Fig. 2:**
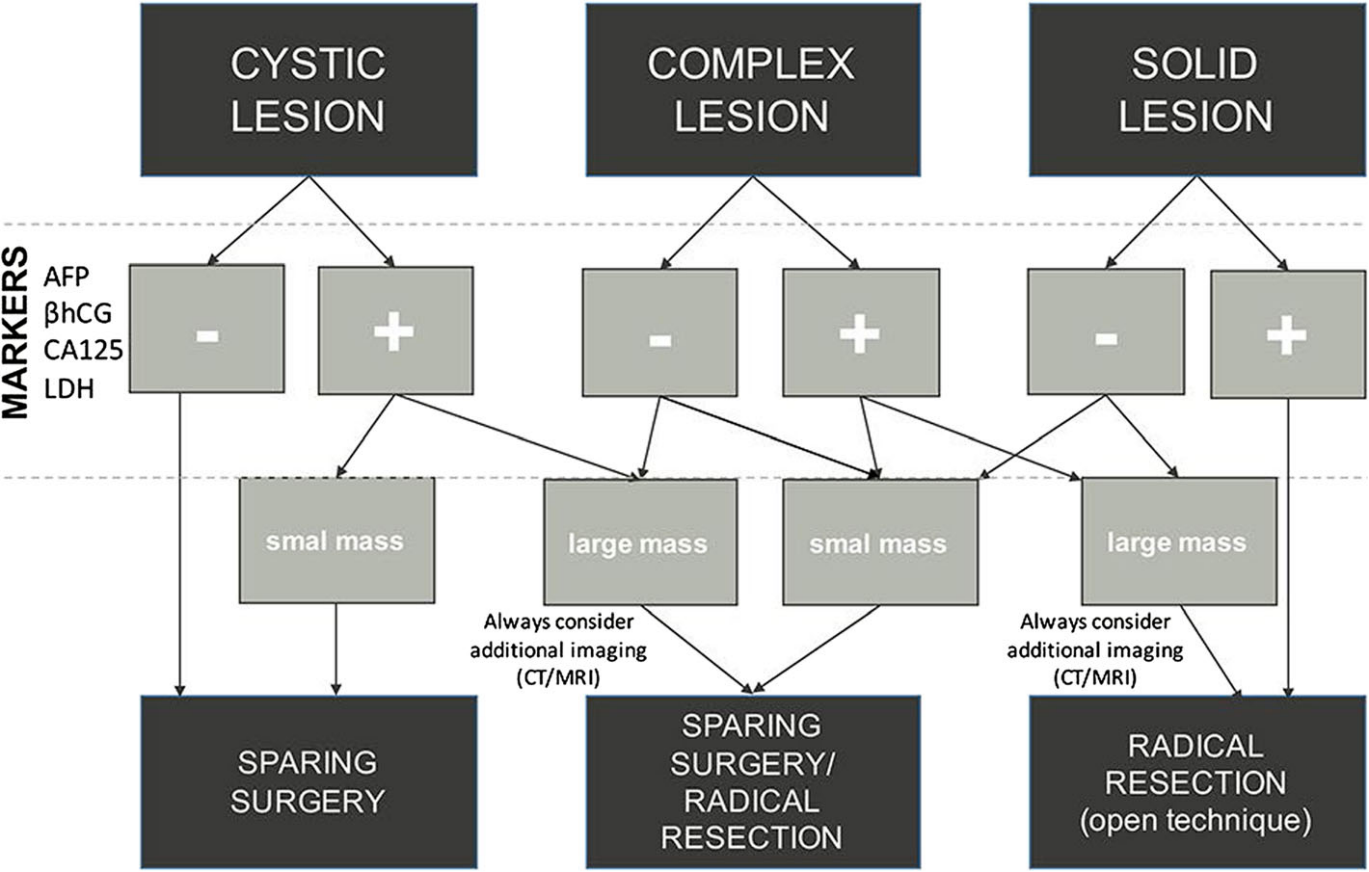
A management algorithm for ovarian lesions in girls

In our material, the overall preservation rate was 38.54%. The preservation rates reported in other series vary between the studies (24-82.9%) [1, 2, 5, 8–10, 14, 15, 29, 30]. There were some reviews indicating lower rates of oophorectomy when a gynecologic surgeon was present [1, 4, 5, 11, 30]. However, the patient population and inclusion criteria in the studies are heterogenous. The information regarding a surgeon’s rationale for performing oophorectomy on benign masses is also exceptionally documented. These factors render comparison of the results between the series difficult. We were unable to evaluate the number of surgeons reporting difficulties in identifying normal ovarian tissue, which could impede preservation attempts. Additionally, patient characteristics and hospital factors might also influence the treatment. The use of the laparoscopic technique was associated with higher preservation of ovarian tissue (55.66% vs 17.44% in laparotomy cases). This corroborates with other authors recommending this method in treatment of tumor-like and benign masses [2, 7, 15]. On the other hand this is a retrospective study and a suspicion of malignant lesion was always an indication for a formal open procedure with preference for unilateral oophorectomy, especially when a lesion was found large. The ovarian-sparing technique has been widely adopted in pediatric surgical centers in girls with benign lesions. However, it requires verification based on a long-term follow-up review [3–5, 9, 14–16, 19, 31]. Similarly, application of minimally invasive procedures in ovarian pathology in girls needs clinical verification although it seems an ideal approach for smaller lesions with no apparent potential for malignancy. Until more prospective clinical trials regarding this topic are conducted, efforts should be made to improve operative techniques and treatment planning with the aim of increasing the rate of ovarian-sparing procedures. Given the above mentioned characteristics of malignant and non-malignant lesions we propose a simplified algorithmic management approach to ovarian masses in girls (Fig. 2). Specific aspects of the surgical procedure itself were not included as it is beyond the scope of this study.

As fertility-sparing procedures are a priority in the constantly aging society, every attempt to improve our knowledge concerning this topic is of great importance. There is no doubt that contemporary medicine should not be a matter of chance. Evidence based medicine is a key to good medical practice and informed decisions. The proposed algorithm is based on a large study group. However, we believe that there is a great need of creating a uniform treatment plan based on the results of the multicenter prospective study and such an initiative is now being introduced in our country. This following study will allow us to verify our concept.

In case of malignant lesions in girls the prognosis is usually excellent, with an early stage and a low grade of the disease diagnosed in this age group [13, 16, 32]. However, ovarian preservation rates decrease with the size of the tumor and many studies indicate that oncologic treatment, including chemotherapy and radiotherapy, increase infertility [33, 34]. This is why it is crucial to diagnose these lesions in their early stages. Unfortunately, our study reveals a significant delay in diagnosis of ovarian malignancy in girls despite their obvious presentation. Nearly 80% of girls with malignant lesions of the ovary presented with an abdominal mass in our material. This issue indicates some degree of negligence by the patients themselves, their parents or some medical practitioners. How to raise awareness among adolescent girls, their parents and healthcare professionals about potential ovarian mass in the case of abdominal enlargement remains a very delicate but important issue.

## Conclusions

Predominantly solid structures noted on imaging studies, large dimension and positive tumor markers are clinical predictors of malignancy. A diagnosis of purely cystic lesions with negative markers or of a small size should be an indication for a gonad-sparing procedure. Treatment guidelines for ovarian lesions in children should be established on the basis of multicenter prospective studies and introduced as soon as possible in order to improve and unify the ovarian preservation rates across the pediatric surgical centers. Efforts should be made to raise public awareness of ovarian masses in girls.

## Abbreviations

AFP: Alpha-fetoprotein
CA125: Cancer antigen 125
CT: Computed tomography
LDH: Lactate dehydrogenase
MRI: Magnetic resonance imaging
US: Ultrasound scan
β-hCG: Beta subunit of human chorionic gonadotropin
